# *Enterobacter* sp. LU1 as a novel succinic acid producer – co‐utilization of glycerol and lactose

**DOI:** 10.1111/1751-7915.12458

**Published:** 2016-12-01

**Authors:** Marcin Podleśny, Piotr Jarocki, Jakub Wyrostek, Tomasz Czernecki, Jagoda Kucharska, Anna Nowak, Zdzisław Targoński

**Affiliations:** ^1^ Department of Biotechnology, Human Nutrition and Food CommoditiesLublin University of Life Sciences8 SkromnaLublin20‐704Poland; ^2^ Department of Analysis and Food Quality AssessmentLublin University of Life Sciences8 SkromnaLublin20‐704Poland

## Abstract

Succinic acid is an important C4‐building chemical platform for many applications. A novel succinic acid‐producing bacterial strain was isolated from goat rumen. Phylogenetic analysis based on the 16S rRNA sequence and physiological analysis indicated that the strain belongs to the genus *Enterobacter*. This is the first report of a wild bacterial strain from the genus *Enterobacter* that is capable of efficient succinic acid production. Co‐fermentation of glycerol and lactose significantly improved glycerol utilization under anaerobic conditions, debottlenecking the utilization pathway of this valuable biodiesel waste product. Succinic acid production reached 35 g l^−1^ when *Enterobacter* sp. LU1 was cultured in medium containing 50 g l^−1^ of glycerol and 25 g l^−1^ of lactose as carbon sources.

## Introduction

In 2004, the US Department of Energy published a report on chemical compounds with the strongest market demand in the near future. This report identified 12 crucial ‘building blocks’ that can be subsequently converted to a number of high‐value bio‐based chemicals or materials. One such building block is succinic acid (Werpy and Petersen, 2004). This dicarboxylic acid has many industrial applications, from the food industry, where it is used as an acidifier, flavour‐enhancing substance or bacteriostatic agent, to its use in a completely biodegradable polyester – polybutylene succinate (PBS) (McKinlay *et al*., 2007). Succinic acid is mainly produced from maleic anhydride (a petroleum‐originating material) by chemical synthesis. Strict emission‐reducing standards and an increasing pursuit of environment‐friendly technologies have spurred interest in the microbiological synthesis of succinic acid (Jansen and Gulik, 2014). *Actinobacillus succinogenes*,* Anaerobiospirillum succiniciproducens*,* Mannheimia succiniciproducens* or *Basfia succiniciproducens* are some of the best studied natural succinic acid producers (Ahn *et al*., 2016). A crucial element of each biotechnological process is a cheap and renewable feedstock, such as glycerine. Producing fuels and reduced chemicals, such as succinic acid, is much more of a redox‐balanced process when conducted on glycerol (due to the high degree of reduction in the carbon atoms) than on more oxidized carbohydrate‐based feedstocks, such as glucose. Moreover, some studies have indicated that microbial production of succinic acid on glycerol minimizes side product formation (Lee *et al*., 2001).

The advantages of glycerol as a feedstock for biotechnological succinic acid production are countered by the difficulty of utilization of glycerol as a sole carbon source under anaerobic conditions by most microorganisms. This metabolic problem can be solved by the addition of external electron acceptors, for example fumaric acid or nitrates (Schindler *et al*., 2014). The application of such electron acceptors, respectively, is not economical (in the case of fumaric acid addition) or increases the formation of side products (in the case of acetic acid) at the expense of succinic acid (Clomburg and Gonzalez, 2013). Until recently, effective glycerol utilization under anaerobic conditions has been considered limited to microorganisms with an active 1,3‐propanediol biosynthetic pathway (Gupta *et al*., 2009) or producing both ethanol and 1,2‐propanediol (Dharmadi *et al*., 2006). Of course, there are microorganisms that can ferment glycerol without forming propanediol products, such as *Propionibacterium acidipropionici* (Himmi *et al*., 2000) and *Anaerobiospirillum succiniproducens* (Lee *et al*., 2001), but these organisms have not been thoroughly investigated. The glycerol metabolic problem can also be overcome by cultivation under aerobic conditions. Such an approach was applied by Yuzbashev *et al*. (2010) using the genetically modified yeast *Yarrowia lipolytica*. The use of yeast in the succinic acid production process has an additional advantage over bacterial‐based technologies because it can be conducted in a low pH environment, which substantially lowers the costs associated with further downstream processes (Lopez‐Garzon and Straathof, 2014).

Of the microorganisms commercially used for succinic acid production, only *B. succiniciproducens* can efficiently utilize glycerol during the fermentation process (Scholten *et al*., 2009). Therefore, a search for new microorganisms utilizing glycerol during succinic acid production process is of the outmost significance as it is expected to broaden the spectrum of microbial biocatalysts capable of efficient utilization of raw materials other than glucose.

In this study, a novel succinic acid‐producing strain isolated from goat rumen is described, including its culture and biochemical characteristics. Physiological and nutritional factors affecting succinic acid production by *Enterobacter* sp. LU1 were investigated. Moreover, the phylogenetic position of this bacterium was characterized based on 16S rRNA, *rpoB* and *gyrB* sequence information. This study is a preliminary step towards the introduction of a novel natural biocatalyst for a succinic acid production process that uses glycerol‐containing fermentation media.

## Results and discussion

### Isolation and identification of a bacterial strain capable of succinic acid production on glycerol as a carbon source

**Figure 1:**
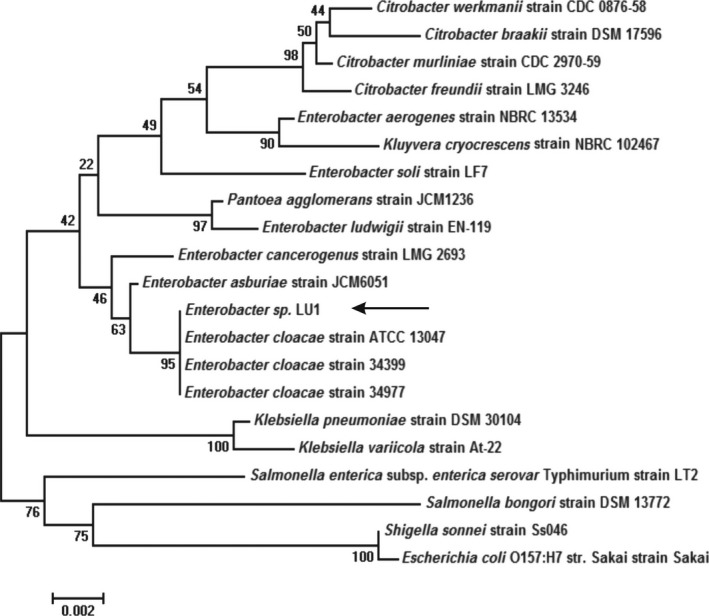
Phylogenetic tree based on partial sequences of 16S rRNA for strain LU1 and different species of the *Enterobacteriaceae* family. The tree was constructed using the neighbour‐joining method from 1000 bootstrapping replicates with the software package mega version 6.0.

The environmental isolation procedure yielded a Gram‐negative rod‐shaped bacterium (1.14 μm (±0.14) in length and 0.75 μm (±0.1) in width) (Fig. S1) that produced approximately 2 g l^−1^ of succinic acid and nearly 0.5 g l^−1^ acetic acid on media containing glycerol. When glucose served as the source of carbon, the main fermentation products were 2,3‐butanediol (4 g l^−1^), ethanol (1.7 g l^−1^), formic acid (1.5 g l^−1^), acetic acid (1.3 g l^−1^) and lactic acid (1.2 g l^−1^), and the concentration of succinic was only approximately 0.7 g l^−1^. Sequencing and analysis of the 16S rRNA gene (GenBank ID: KU499554) revealed that the closest relatives of strain LU1 were *Enterobacter cloacae* strain 34977 and *E. cloacae* strain 34399 (both with 100% similarity to the novel isolate) (Fig. 1). Bacterial species belonging to the *E. cloacae* complex are closely related, which often leads to misidentification when traditional methods are used (Hoffmann and Roggenkamp, 2003). Therefore, additional gene analysis was performed, and the nucleotide sequences of polymerase beta subunit *rpoB* (GenBank ID: KU499555) and gyrase B subunit *gyrB* GenBank ID: KU499556) were obtained. Based on its response to the API 20E test, the isolated bacterial strain was identified as a strain of *E. cloacae* with 95.1% identification. The results of the API 50CH tests of the newly isolated bacterium are presented in Table S3. From a biochemical perspective, *Enterobacter* sp. LU1 appears to be more closely related to *Enterobacter hormaechei* subsp. *steigerwaltii* than to the other members of the *E. cloacae* complex (based on fermentation of D‐adonitol or D‐arabitol, for example) (Hoffmann *et al*., 2005). Based on these results and on the sequence similarity and phylogenetic analyses, the newly isolated bacterial strain LU1 was assigned to the *E. cloacae* complex, and its closest relative is probably *E. hormaechei* subsp. *steigerwaltii*.

### Glycerol utilization under anaerobic conditions and co‐fermentation strategy for debottlenecking this pathway

**Figure 2:**
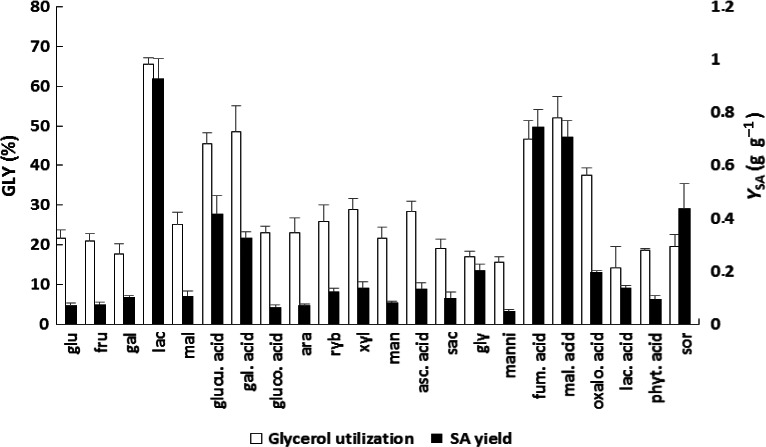
Co‐fermentation of various carbon sources by *Enterobacter* sp. LU1 to enhance the production of succinic acid. *glu* – glucose; *fru* – fructose; gal – galactose; *lac* – lactose; *mal* – maltose; *glucu. acid* – glucuronic acid; *gal. acid* – galacturonic acid; *gluco. acid* – gluconic acid; *ara* – arabinose; *ryb* – ribose; *xyl* – xylose; *man* – mannose; *asc. acid* – ascorbic acid; *sac* – saccharose; *gly* – glycerol; *manni* – mannitol; *fum. acid* – fumaric acid; *mal. acid* – malic acid; *oxalo. acid* – oxaloacetic acid; *lac. acid* – lactic acid; *phyt. acid* – phytic acid; *sor* – sorbitol. Succinic acid (SA); glycerol (GLY). Percentage of glycerol utilization (□); overall succinic acid yield (calculated as gram product (succinate)/gram substrate(glycerol and lactose) consumed) (■). The cultivations were conducted at 37°C in tightly capped 20 ml serum bottles with 8 ml of medium and stirring at 160 rpm. Data are the means ± SDs of three parallel experiments.

Although glycerol in the fermentation medium was indispensable to exceed 0.4 g l^−1^ succinic acid production by *Enterobacter* sp. LU1, further increase in the concentration of glycerol had little effect on the extent of succinate formation. Moreover, higher glycerol concentrations in the fermentation medium reduced the percentage conversion of this substrate by *Enterobacter* sp. LU1. The maximum succinic acid concentration (1.9 g l^−1^) was obtained at a glycerol concentration of 15 g l^−1^ (Fig. S2). In summary, glycerol was hardly metabolized under anaerobic conditions, which resulted in low succinic acid yields. The results of the co‐fermentation studies revealed the maximal positive effect of lactose addition as a co‐substrate for the culture grown on glycerol (% consumption of glycerol at 65 and Y_SA_ reaching almost 0.93) (Fig. 2). The addition of lactose resulted in superior glycerol utilization compared to the addition of fumaric acid (46% glycerol consumption and Y_SA_ = 0.75) or malic acid (52% glycerol consumption and Y_SA_ = 0.7). The addition of glucose resulted in a characteristically low succinic acid yield and glycerol utilization, probably due to catabolic repression, which blocks alternative catabolic pathways in the presence of glucose (Saier and Roseman, 1976). Kamzolova *et al*. (2011) reported that glucose is a strong repressor of hexadecane metabolism and a mild repressor of oleic acid metabolism (and probably also the metabolism of other fatty acids). By contrast, glycerol does not suppress the metabolism of fatty acids in *Y. lipolytica* yeast grown on a mixture of free saturated fatty acids and glycerol (Papanikolaou *et al*., 2006) or in *Y. lipolytica* cultivated on rapeseed and sunflower oils (Kamzolova *et al*., 2005, 2011). The improvement in glycerol utilization in the presence of fumaric acid was thoroughly described in previous studies on glycerol fermentation (Ryu *et al*., 1998; Na Rhie *et al*., 2014). Malic acid, which is a direct metabolic precursor of fumaric acid in bacterial metabolism, also improved glycerol utilization. Notable improvements in glycerol consumption were observed upon the addition of glucuronic acid (45% glycerol consumption and Y_SA_ = 0.42) and galacturonic acid (48% glycerol consumption and Y_SA_ = 0.33); these improvements are similar to those obtained with the two aforementioned acids (fumaric and malic). This comparison, however, indicates the superiority of fumaric and malic acid addition with regard to the specific production of succinic acid (Y_SA_), which is likely attributable to the more oxidized character of these compounds compared to glucose (Clomburg and Gonzalez, 2013), which can partially compensate for the highly reduced glycerol compounds in nature (Hadiati *et al*., 2014). According to the auxiliary concept of Babel (2009), the simultaneous consumption of physiologically similar substrates can increase the yield of a target product. Morgunov and Kamzolova (2015) reported that *Y. lipolytica* grown on glycerol derived from biodiesel and composed of two substrates (glycerol and fatty acids) displayed the highest biomass yield (Y_x/s_) of 1.05 g g^−1^ and a maximum specific growth rate (μmax) of 0.352 ч^−1^, whereas cells cultivated on pure glycerol or fatty acids displayed biomass yields (Y_x/s_) of 0.47 and 0.87 g g^−1^ respectively (Kamzolova *et al*., 2011). The simultaneous utilization of methanol and glucose by *Hansenula polymorpha* resulted in an increase in biomass yield of up to 25% and a higher growth rate compared to those observed on single substrates (Müller *et al*., 1983). Workman *et al*. (2013) reported that simultaneous utilization of glucose and glycerol resulted in maximum metabolism by *Y. lipolytica* with regard to available oxygen promoting ATP generation by oxidative phosphorylation and increased the specific growth rate and biomass yield. The addition of the other co‐substrates increased glycerol consumption to a maximum of 30%, except oxalocetic acid, which enabled glycerol consumption of approximately 38% accompanied by a low specific productivity of succinic acid (Y_SA_ = 0.19) (Fig. 2).

In summary, the chemical structure of lactose may be responsible for the maximum yield of succinic acid during the co‐fermentation of glycerol. Cleavage of the β‐galactosidase bond within lactose provides hexoses, which can be used for cell mass formation without generating the excess of reducing equivalents, in contrast to glycerol as the only carbon source (Clomburg and Gonzalez, 2013). Moreover, lactose does not trigger catabolic repression and allows the glycerol utilization pathway to function. Therefore, under these conditions, some type of functional separation of carbon source utilization may exist in which glycerol is used principally for energy‐generating pathways ending mainly with reduced products (e.g. succinic acid and ethanol) and is used infrequently to generate structural precursors for biomass synthesis (Schindler *et al*., 2014). Further investigation at the molecular level is required to support these observations.

An essential step towards the use of lactose as a biotechnological feedstock is the identification of a cheap natural product containing this disaccharide. One candidate product is cheese whey. Attempts at producing succinic acid using whey have been described for *A. succinogenes* (Wan *et al*., 2008). The relatively high price per tonne of this substrate (795 $/tonne) compared to glycerol (219 $/tonne) is mainly due to a growing demand for whey proteins (Tan *et al*., 2014). Of economic significance, the process of whey proteins isolation results in the removal of products such as lactose from raw whey and in the formation of permeate (Wong and Hartel, 2014), which alone may be successively applied in the biotechnological production of succinic acid due to its high content of lactose (70–80% (w/w)). Moreover, the liquid whey permeate generated in dairy plants could be (after partial concentration) directly applied for the preparation of a production medium for the exploited biocatalysts, thus reducing the expenses linked with permeate pulverization. Localization of succinic acid production near dairy processing plants producing whey permeate would significantly reduce the costs of transport of this substrate in liquid form.

### Influence of fermentation conditions on succinic acid production by *Enterobacter* sp. LU1

Little effect of altering the weight ratio of co‐substrates on succinic acid concentration was observed after a 96 h cultivation of *Enterobacter* sp. LU1 (approximately 13 g l^−1^) (Fig. S3). In all co‐fermentations, the succinic acid concentration was higher compared with the experiments with glycerol alone (1.2 g l^−1^) or when only lactose was present in fermentation medium (9.5 g l^−1^). Interestingly, lactose concentrations of > 10 g l^−1^ permitted faster succinic acid production in the first period of fermentation, which resulted in the highest succinic acid concentration (11.9 g l^−1^) after 48 h. To analyse the consumption of carbon sources and additional product formation during co‐fermentation, a glycerol‐to‐lactose weight ratio of 15/15 was used (Fig. S4). For the first 12 h, succinic acid production was nearly undetectable (approximately 0.15 g l^−1^), but biomass doubled to 0.94 g l^−1^. After 12 h, intensification of succinic acid production was observed, and the bacterial biomass continued to increase, resulting in 1.4 g l^−1^ dry weight in 24 h. Substrate utilization was also more apparent after 12 h of fermentation (until 48 h at approximately 0.27 g l^−1^ h^−1^) and continued at slower rate (approximately at 0.1 g l^−1^ h^−1^) until 72 h. Then, glycerol consumption ceased, reaching concentration of 3 g l^−1^, and lactose utilization continued, ending at rate of 0.09 g l^−1^ h^−1^. Succinic acid was not the only product of glycerol–lactose co‐fermentation by *Enterobacter* sp. LU1. In addition to this dicarboxylic acid ethanol, acetic acid and formic acid were also produced, and at the end of fermentation, their concentrations reached nearly 2 g l^−1^ (ethanol; acetic acid) and 0.9 g l^−1^ (formic acid).

**Figure 3:**
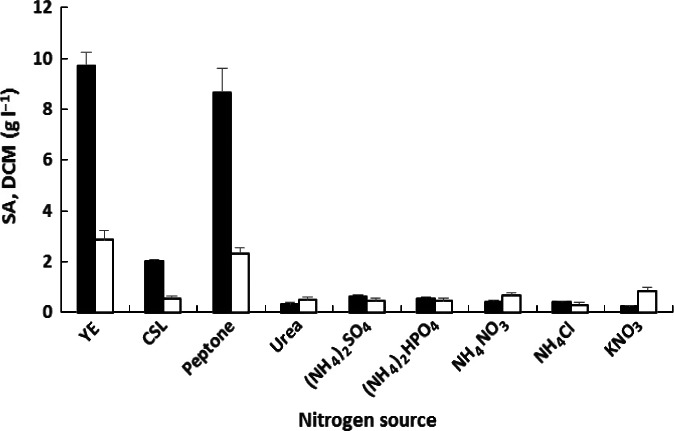
Effects of nitrogen source on succinic acid production and cell growth after 96 h of fermentation. The nitrogen source concentration was 0.5 g l^−1^. Succinic acid (SA) (■); dry cell mass (DCM) (□); yeast extract (YE); corn steep liquor (CSL). Fermentations were conducted in tightly capped 20 ml serum bottles with 8 ml of medium and stirring at 160 rpm. Data are the means ± SDs of three parallel experiments.

Among the various nitrogen sources tested, yeast extract maximally enhanced the production of both succinic acid (9.69 g l^−1^) and cell biomass (2.85 g l^−1^) (Fig. 3). This observation is consistent with the results of previous studies addressing microbiological production of succinic acid using other biocatalysts (Guettler *et al*., 1996a; Okino *et al*., 2008; Song *et al*., 2008). In some cases, the presence of yeast extract is indispensable for fermentative glycerol utilization during succinic acid production (Lee *et al*., 2000). The addition of peptone resulted in only slightly lower succinic acid concentrations (8.65 g l^−1^), indicating that this nitrogen source is suitable for efficient succinic acid production (Schröder *et al*., 2010). Surprisingly, corn steep liquor supplementation resulted in fourfold reduction in succinic acid concentration (2.01 g l^−1^) compared to the two previous organic nitrogen sources. Therefore, the efficacy of corn steep liquor as a suitable nitrogen source for *Enterobacter* sp. LU1 was not confirmed under the tested conditions, contradicting the results of some previous studies (Agarwal *et al*., 2006; Xi *et al*., 2013; Shen *et al*., 2016). The presence of glucose (up to 10% (w/w)) in corn steep may therefore have a negative effect on the level of succinic acid production in the glycerol–lactose system (due to increased catabolite repression). This is likely why low concentrations of succinic acid were obtained in the cultures with corn steep liquor as a source of nitrogen. All tested inorganic nitrogen sources were rather inefficient under the tested conditions (20 ml serum bottles with 8 ml of fermentation medium).

An important factor inevitably connected with bacterial succinic acid production is the use of an appropriate neutralizing agent. The highest succinic acid concentration was observed during 48 h fermentation with MgCO_3_ (2.6 g l^−1^) (Fig. S5a), similar to results obtained for *A. succinogenes* and *B. succiniciproducens* (Guettler *et al*., 1996b; Schröder *et al*., 2010). Further investigations focused on the selection of the optimal concentration of magnesium carbonate. The dry cell mass and concentration of succinic acid increased with magnesium carbonate concentrations up to 25 g l^−1^ and then decreased slowly (Fig. S5b). The production of succinic acid increased significantly when magnesium carbonate was present in the fermentation medium at a minimum concentration 5 g l^−1^, probably due to the pH buffering capacity of magnesium carbonate. Magnesium ions released from this carbonate salt during organic acids production neutralized them and made decreased their detrimental effects on bacterial cells. Figure S5c shows pH values of the fermentation media before and after the fermentation period. Addition of 5 g l^−1^ magnesium carbonate increased the pH value of the fermentation medium to approximately 7.00 and resulted in a succinic acid concentration that was approximately seven times higher (5 g l^−1^) at the end of fermentation in comparison with samples without magnesium carbonate. For reactions with higher magnesium carbonate concentrations (> 5 g l^−1^), there were slight differences in the starting pH values, which oscillated near 8.00. End‐point pH values increased as the magnesium carbonate concentration increased. These results indicate the importance of maintaining the pH of the fermentation medium above 6.00 for the highest succinic acid concentrations. Thus, magnesium carbonate is a crucial factor promoting the production of succinic acid in glycerol and lactose medium by the LU1 strain, but a high initial concentration of the neutralizing agent would negatively affect LU1 metabolism.

Because biocatalytic processes are also temperature‐dependent, cell growth and succinic acid production by *Enterobacter* sp. LU1 were also examined as a function of temperature (Fig. S6). A temperature of 34°C was most favourable for succinic acid production because it enabled eightfold greater production than that observed at 27°C. By contrast, cell growth of *Enterobacter* sp. LU1 was virtually unchanged (approximately 2 g l^−1^) throughout the temperature range of 27–34°C. A temperature of 37°C appears to be suboptimal both for succinic acid concentration and for cell biomass. At 40°C, dramatic decrease was observed in both succinic acid concentration (nearly 10‐fold) and cell biomass (tenfold). In a previous study, succinic acid was produced by genetically modified *Enterobacter aerogenes* (Tajima *et al*., 2014) at 34°C, whereas for *Corynebacterium glutamicum* strains, temperatures between 30 and 33°C were often employed (Okino *et al*., 2008; Wang *et al*., 2014). In most cases, bacterial succinic acid production has been conducted at 37–40°C (Chen *et al*., 2014; Gunnarsson *et al*., 2014), and for yeast‐based process, the commonly chosen temperatures were at 28–30°C (Raab *et al*., 2010).

### Bioreactor cultivation of *Enterobacter* sp. LU1

**Figure 4:**
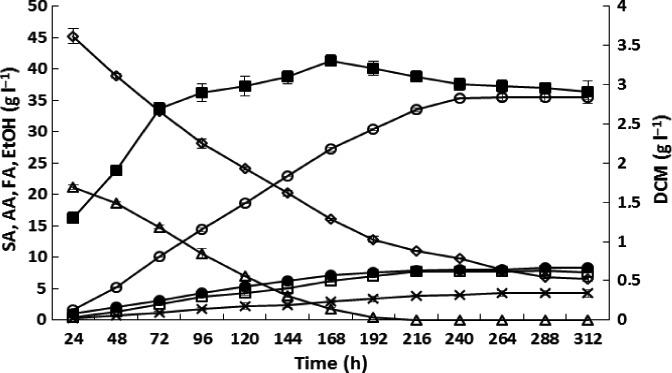
Enhancement of succinic acid production using glycerol (50 g l^−1^) and lactose (25 g l^−1^) as sources of carbon in a 2 litre fermenter (34°C; stirring at 250 rpm). The pH was maintained at 7 throughout the cultivation using a solution consisting of 5% NaOH (w/v) and 20% Na_2_CO_3_ (w/v). Dry cell mass (DCM) (■); succinic acid (SA) (○); glycerol (GLY) (◊); lactose (LAC) (Δ); acetic acid (AA) (□); formic acid (FA) (×); ethanol (EtOH) (●). The values are the means of two independent samples.

Although the capability of environmental strains of *Enterobacter* to produce succinic acid as the main product during anaerobic conditions has not been described in the literature, the results obtained in this study are not competitive with those for other thoroughly described microbial producers of succinic acid (Beauprez *et al*., 2010). One way to increase bioproduct concentration during fermentation is by precisely controlling fermentation conditions, which can be achieved using different bioreactors (Ren *et al*., 2013; Yan *et al*., 2014). This study was initially unable to achieve succinic acid concentrations higher than 13 g l^−1^. Application of a 2 l stirred bioreactor allowed a maximum succinic acid concentration of 35 g l^−1^ to be achieved in 312 h (Fig. 4). The high succinic acid concentration could be reached mainly because of the separate sterilization of lactose, YE and magnesium carbonate. Previous fermentations performed in our laboratory were conducted after collective sterilization of carbon and nitrogen sources along with a neutralizing agent. Such an approach probably promoted the formation of Maillard compounds, which can negatively affect the growth of microbial cells (Ames, 1992) and likely resulted in dramatic decrease in productivity and final succinic acid concentrations. During fermentation, simultaneous utilization of lactose and glycerol was observed until 192 h, when the lactose concentration approached 0 g l^−1^. Glycerol was not consumed completely even after 312 h (approximately 6 g l^−1^ remained). The total concentration of side products was approximately 21 g l^−1^, and the side products included ethanol (8.35 g l^−1^), acetic acid (7.61 g l^−1^) and formic acid (4.25 g l^−1^). The maximum cell concentration increased until 72 h and slightly decreased after reaching 3.31 g l^−1^. From 168 h onward, we observed a reduction in the biomass concentration, probably due to the ageing of bacterial cells and their lysis. Succinic acid production apparently remained at a similar level during fermentation until 216 h, after accounting for the arrest of biomass growth. The succinic acid concentrations obtained with *Enterobacter* sp. LU1 co‐fermentation were similar to those obtained with other natural succinic acid producers cultivated on glycerol but were nearly 35% smaller than those obtained with *B. succiniciproducens* DD1 cultivated on glycerol with maltose (Table 1). Overall yield and productivity were lower for *Enterobacter* sp. LU1 (Y_SA_ = 0.51; productivity = 0.11) compared with competitive bacterial strains. The most similar results were those for *A. succinogenes* 130Z, which were nearly 2 and 1.5 times higher respectively (Y_SA_ = 0.96; productivity = 0.139).

**Table 1:** Comparison of SA production on glycerol by various native bacterial species

Strain	Fermentation conditions	Concentration (g l^−1^)	Yield (g g^−1^)	Productivity (g l^−1^ h^−1^)	Reference
*Actinobacillus succinogenes* 130Z	Microaerobic, Continuous	31.7	0.96	0.139	Schindler *et al*., 2014
*Anaerobiospirillum succiniciproducens* ATCC 53488	Anaerobic, Fed‐batch (feeding with YE and glycerol)	19	1.6	0.157	Lee *et al*., 2001
*Basfia succiniciproducens* DD1	Anaerobic, Batch	19.5	1.12	0.81	Schröder *et al*., 2010
*B. succiniciproducens* DD1	Anaerobic, Batch (maltose addition)	53.2	1.01	2.21	Schröder *et al*., 2010
*Enterobacter* sp. LU1	Anaerobic, Batch (lactose addition)	35	0.51	0.11	This study

The proposed method of succinic acid production is not yet competitive with the best technologies utilizing genetically modified yeast or *E. coli*. However, this work extends the list of potential biocatalysts that can be feasibly applied to utilize waste glycerol in the process. Continued research may further improve the competitiveness of the described biotechnological system using *Enterobacter* sp. LU1 particularly regarding the final concentration of succinic acid obtained in the post‐culture liquid.

## Experimental procedures

### Cultivation of *Enterobacter* sp. LU1

The strain was maintained frozen at −80°C with 20% (w/w) glycerol added. Inoculum cultures were grown anaerobically at 37°C in 100 ml serum bottles with a 50 ml working volume capped with gas‐tight butyl rubber stoppers in the brain heart infusion medium (BHI) (Oxoid, UK), pH 7.4. Overnight cultures were used to inoculate the fermentation medium (10% (v/v)). Prior to inoculation, the overnight cultures were centrifuged, and the bacterial biomass obtained was suspended in a vitamin mix solution (Table S4). The vitamin mix with bacterial cells was added to the fermentation medium at 2.5 ml l^−1^. The fermentation medium consisted of (g l^−1^): carbon source 0–30; yeast extract 10; MgCO_3_ 20; NaCl 1; Na_2_HPO_4_ 0.78; NaH_2_PO_4_ 1.16; MgCl_2_ × 6H_2_O 0.2; CaCl_2_ × 2H_2_O 0.2; and distilled water. Unless otherwise stated, the initial pH of the medium was 7.4–8.0, depending on the magnesium carbonate concentration, and the temperature for batch growth was maintained at 37°C. The cell concentration, soluble product composition and substrate utilization were typically monitored after a 96 h cultivation conducted on a rotary shaker at 160 rpm in 20 ml serum bottles (working volume 8 ml) with gas‐tight butyl rubber stoppers (CO_2_ was the headspace gas in all serum bottle incubations (about 0.8 bar overpressure)). Culture purity was ensured by routine examination under light microscope during end‐point samples withdrawal (simple staining with crystal violet) and by plating on BHI agar medium. Strain purity was also checked before starting incubations on BHI agar medium. Plates were incubated in both an aerobic and anaerobic conditions.

### Description of experiments

After the isolation of *Enterobacter* sp. LU1, the effects of different physiological and nutritional parameters on succinic acid production were studied. The first parameter considered was the glycerol concentration (0–30 g l^−1^) because the strain produced succinic acid on the medium containing glycerol during the isolation and screening procedure (approximately 2 g l^−1^ SA). In another set of experiments, glycerol was supplemented with different substrates (Table S2). Fermentations were conducted at a glycerol concentration of 20 g l^−1^ and with 10 g l^−1^ additional carbon source. To investigate the effect of glycerol and lactose as a mixed carbon source on succinic acid production, five weight ratios of glycerol to lactose were chosen (15/0; 10/20; 15/15; 20/10; and 0/15). Once equivalent concentrations of glycerol and lactose were established as optimal (15 g l^−1^), the best nitrogen source for succinic acid production was investigated. The influences of inorganic ((NH_4_)_2_SO_4_; (NH_4_)_2_HPO_4_; NH_4_NO_3_; NH_4_Cl; KNO_3;_ and urea) and organic nitrogen sources (yeast extract (BTL, Poland); peptone (BTL, Poland); and corn steep liquor (Sigma‐Aldrich, USA) were determined at the same level of nitrogen, 0.5 g l^−1^. The organic carbon load of N sources was not normalized during fermentations. Because conducting succinic acid fermentation in neutral conditions requires appropriate neutralizing agent usage, four different carbonate salts (CaCO_3_, MgCO_3_, Na_2_CO_3_ and NaHCO_3_) were investigated, all at the same concentration of 5 g l^−1^. Next, the optimal concentration of magnesium carbonate was studied in the range of 0–60 g l^−1^. Finally, the effect of temperature on succinic acid production was studied (27–44°C). For the bioreactor cultivations, the pre‐culture of *Enterobacter* sp. LU1 (200 ml) was inoculated into a Biostat A fermenter (Sartorius AG, Germany) containing 2 l of fermentation medium. The fermentation temperature was 34°C with stirring at 250 rpm. The pH value was adjusted automatically with solution of 5% NaOH (w/v) and 20% Na_2_CO_3_ (w/v). The initial substrate concentrations were 50 g l^−1^ glycerol and 25 g l^−1^ lactose; the solutions of the substrates were sterilized separately from the solution containing the nitrogen source. The other constituents of the medium were as follows (g l^−1^): YE 15; MgCO_3_ 5; Na_2_HPO_4_ 0.31; NaH_2_PO_4_ × H_2_O 1.16; MgCl_2_ × 6H_2_O 0.2; CaCl_2_ 0.15; and NaCl 1. The succinate yield was calculated as gram product (succinate)/gram substrate(glycerol and lactose) consumed.

### Analytical methods

Cell growth was monitored by measuring the absorbance of the broth at 600 nm (OD600) after diluting the sample 1:1 with 7% HCl (v/v). The biomass concentration of the *Enterobacter* sp. LU1 strain was estimated by determining the dry cell weight (DCW) using a predetermined correlation curve obtained between the absorbance measured at 600 nm and the cell dry weight (g l^−1^). Samples for succinic acid and by‐product detection were prepared by centrifugation of the culture broth at 6000 × g for 5 min. The resulting supernatant, after dilution with water (1:1), was analysed by high‐performance liquid chromatography system (Gilson) equipped with an ion exchange column (Aminex HPX‐87H, Bio‐Rad) and a refractive index detector using 0.03 M sulfuric acid as mobile phase at 42°C (Dharmadi *et al*., 2006).

### Statistical analysis

The data were analysed using Microsoft Excel 2013 (Microsoft Corporation) and statistica software (version 8.0; StatSoft, www.statsoft.com) using an ANOVA procedure for analysis of variance and Tukey's test for ranking the means.

## Conflict of Interest

None declared.

## Supporting information


**Fig. S1**. Light microscope micrograph of strain LU1 stained with crystal violet.
**Fig. S2**. Effect of different concentrations of glycerol on succinic acid production and cell growth from Enterobacter sp. LU1 after 96h of incubation.
**Fig. S3**. Effect of the weight ratio of glycerol to lactose on succinic acid production.
**Fig. S4**. Time course of batch fermentation of glycerol (15 gl‐1) and (15 gl‐1).
**Fig. S5**. (A) Effects of dicarbonate salt (5 g l‐1) on succinic acid production and cell growth after 48h of incubation and (B) influence of magnesium carbonate concentration on succinic acid concentration and cell growth after 96h of fermentation with (C) pH values of microbiological media before and after 96 h fermentation.
**Fig. S6**. Effects of temperature on cell growth and succinic acid production.
**Table S1**. Sequences of oligonucleotide primers used in this study.
**Table S2**. Co‐substrates tested in fermentation studies with glycerol.
**Table S3**. Biochemical reactions of Enterobacter sp. LU1 in the API 50CHE system after 48h of incubation.
**Table S4**. Vitamin mix solution composition.
