# Supplementary material for: Enterobacter sp. LU1 as a novel succinic acid producer – co‐utilization of glycerol and lactose
Source: Microb Biotechnol. 2016 Dec 1;10(2):492–501. doi: 10.1111/1751-7915.12458 (PMC5328818; doi:10.1111/1751-7915.12458)
Supplement: Supplementary file 1 — Fig. S1. Light microscope micrograph of strain LU1 stained with crystal violet. Fig. S2. Effect of different concentrations of glycerol on succinic acid production and cell growth from Enterobacter sp. LU1 after 96h of incubation. Fig. S3. Effect of the weight ratio of glycerol to lactose on succinic acid production. Fig. S4. Time course of batch fermentation of glycerol (15 gl‐1) and (15 gl‐1). Fig. S5. (A) Effects of dicarbonate salt (5 g l‐1) on succinic acid production and cell growth after 48h of incubation and (B) influence of magnesium carbonate concentration on succinic acid concentration and cell growth after 96h of fermentation with (C) pH values of microbiological media before and after 96 h fermentation. Fig. S6. Effects of temperature on cell growth and succinic acid production. Table S1. Sequences of oligonucleotide primers used in this study. Table S2. Co‐substrates tested in fermentation studies with glycerol. Table S3. Biochemical reactions of Enterobacter sp. LU1 in the API 50CHE system after 48h of incubation. Table S4. Vitamin mix solution composition. [file MBT2-10-492-s001.pdf]

SUPPORTING INFORMATION (SI)

Enterobacter sp. LU1 as a novel succinic acid producer – co-utilization of glycerol and lactose.

Marcin Podleśny[1]*, Piotr Jarocki[1], Jakub Wyrostek[2], Tomasz Czernecki[1], Jagoda Kucharska[1], Anna Nowak[1], Zdzisław Targoński[1]

[1]Department of Biotechnology, Human Nutrition and Food Commodities, Lublin University of Life Sciences, 8 Skromna, 20-704 Lublin, Poland

[2]Department of Analysis and Food Quality Assessment, Lublin University of Life Sciences, 8 Skromna, 20-704 Lublin, Poland

*corresponding author, e-mail:podlesnymarcin@hotmail.com

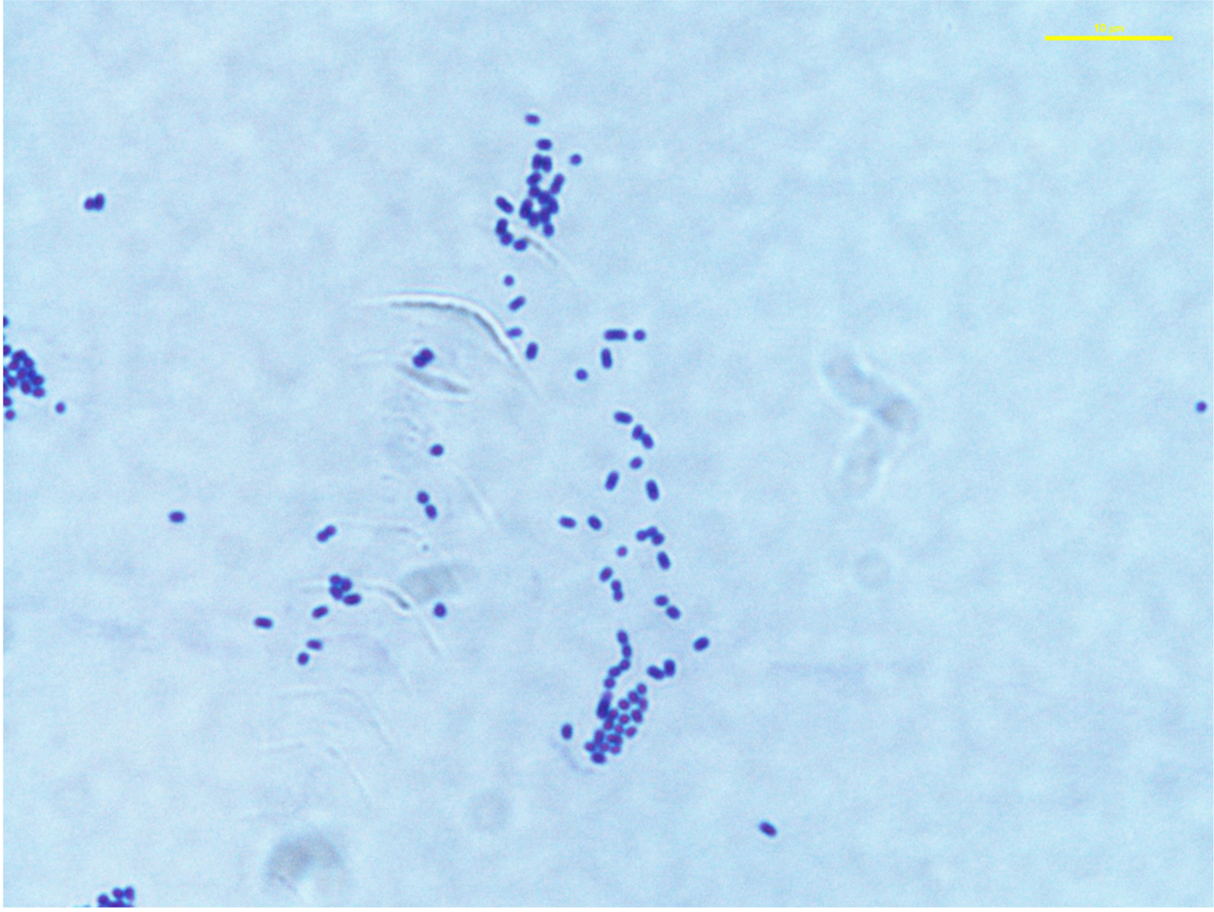

Figure S1. Light microscope micrograph of strain LU1 stained with crystal violet. Micrograph made with Nikon Eclipse 90i microscope equipped with NIS Elements AR microscope imaging software. Cells grown anaerobically on BHI agar. Bar, 10 μm.

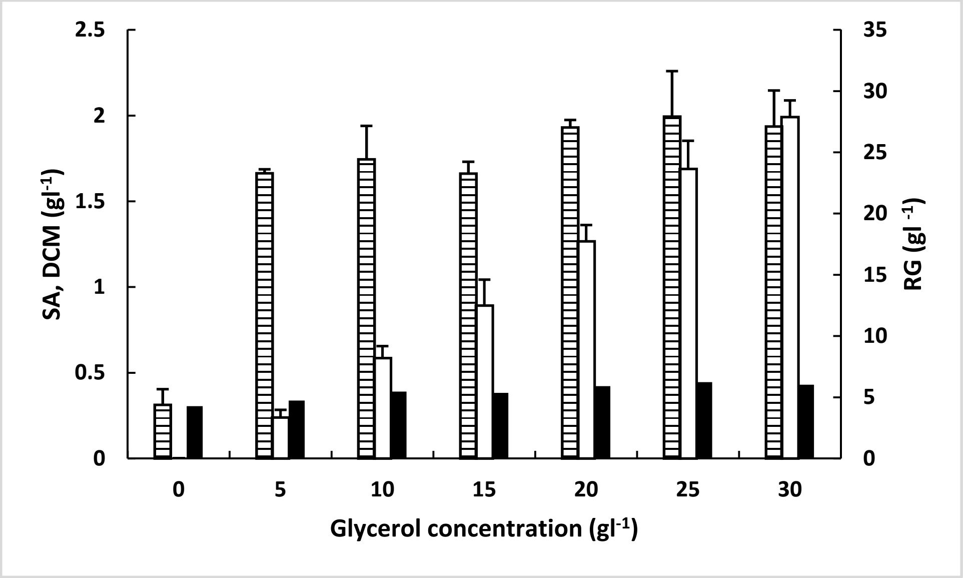

Figure S2. Effect of different concentrations of glycerol on succinic acid production and cell growth from *Enterobacter* sp. LU1 after 96h of incubation. Succinic acid (SA) (≡); Dry Cell Mass (DCM) (■); Residual glycerol (RG) (□). Data are the means ±SDs from three parallel experiments. The fermentations were carried out at 37 °C in tightly capped 20-ml serum bottles, under stirring at 160 rpm. Data are the means ±SDs from three parallel experiments.

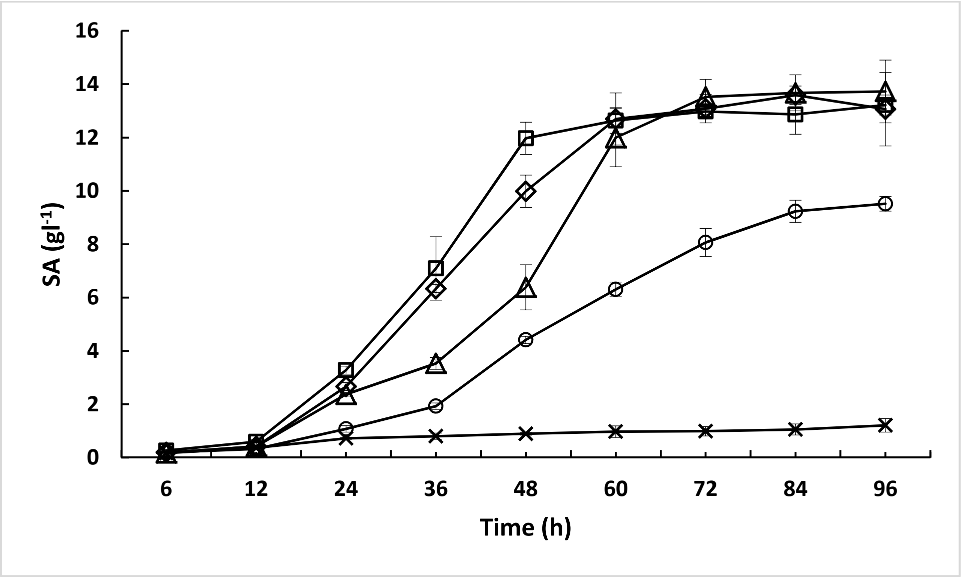

Figure S3. Effect of the weight ratio of glycerol to lactose on succinic acid production. Glycerol/lactose weight ratio: G10/L20 (□); G15/L15 (◊); G20/L10 (Δ); G15 (×); L15 (○).The fermentations were conducted at 37 °C in tightly capped 20-ml serum bottles with 8 ml of medium and stirring at 160 rpm. Data are the means ± SDs of three parallel experiments.

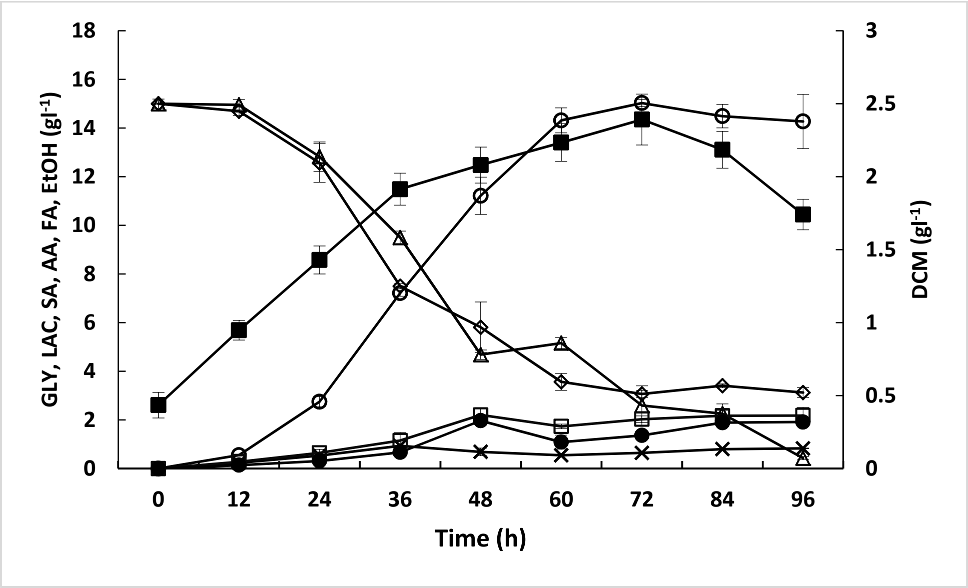

Figure S4. Time course of batch fermentation of glycerol (15 gl^{-1}) and lactose (15 gl^{-1}). Dry Cell Mass (DCM) (■); succinic acid (SA) (○); glycerol (GLY) (◊); lactose (LAC) (Δ); acetic acid (AA) (□); formic acid (FA) (×); ethanol (EtOH) (●). The fermentations were conducted at 37 °C in tightly capped 20-ml serum bottles with 8 ml of medium and stirring at 160 rpm. Data are the means ± SDs of three parallel experiments.

a)

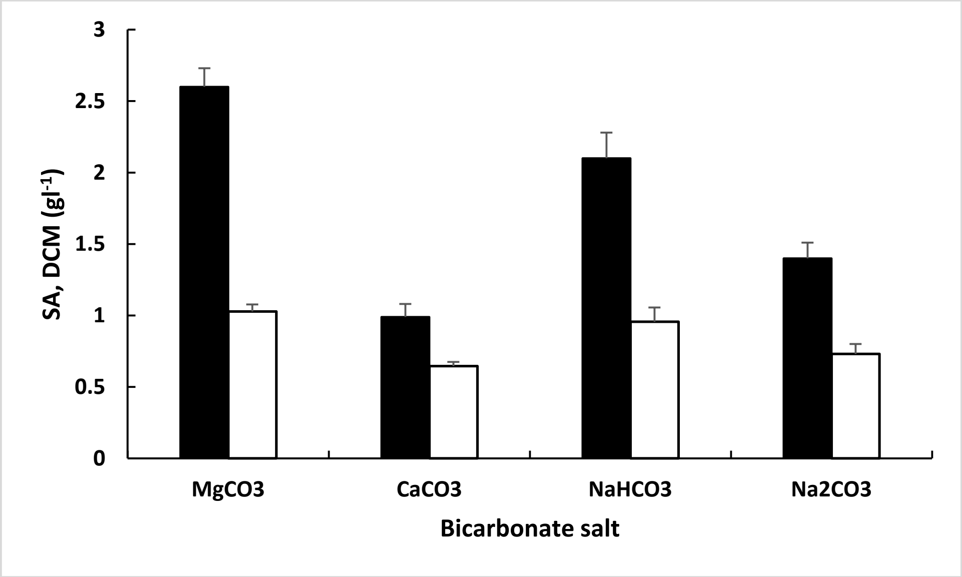

SA, DCM (gl-1)
3
2.5
2
1.5
1
0.5
0
MgCO3
CaCO3
NaHCO3
Na2CO3
Bicarbonate salt

b)

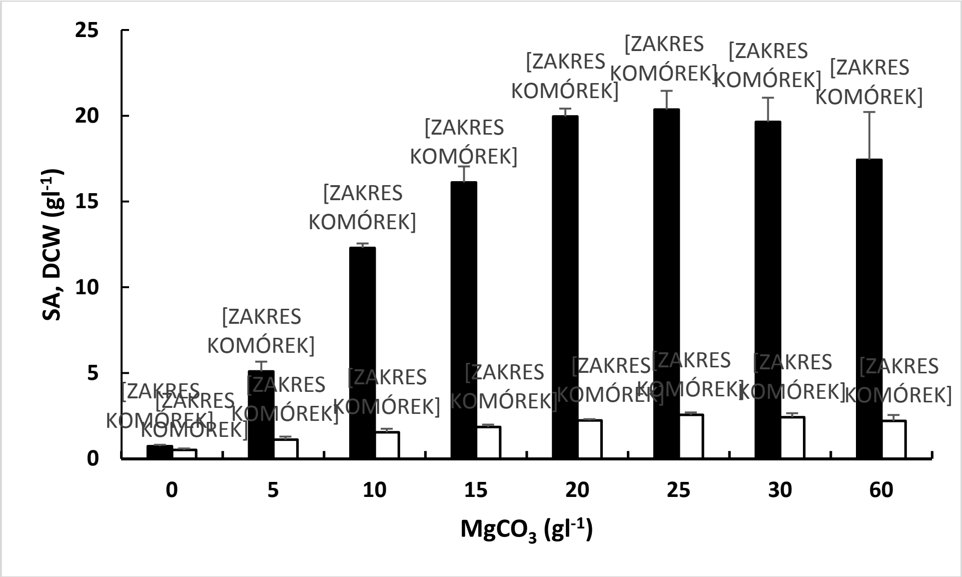

SA, DCW (gl-1)
25
20
15
10
5
0
[ZAKRES KOMÓREK]
0
5
10
15
20
25
30
60
MgCO3 (gl-1)

c)

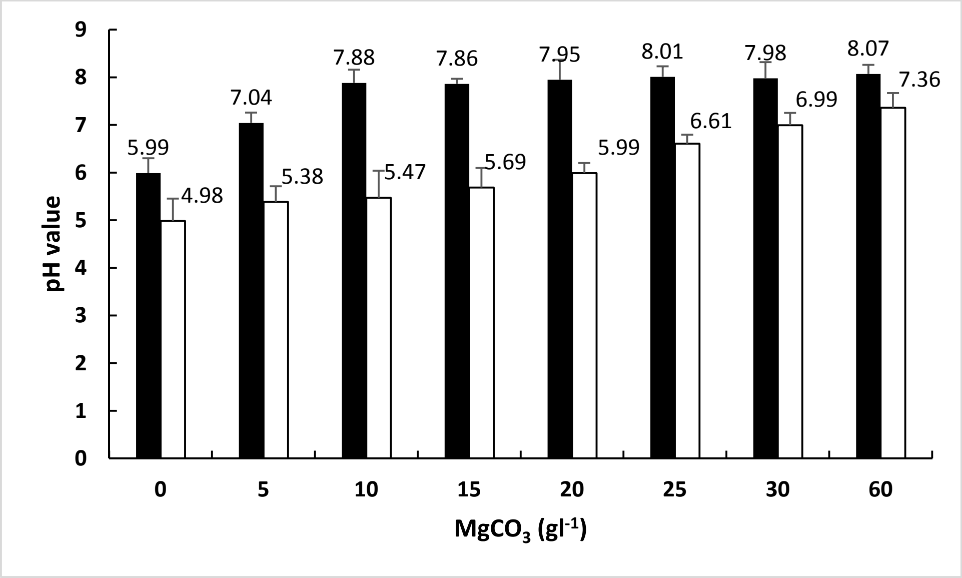

Figure S5. (A) Effects of dicarbonate salt (5 g l^{-1}) on succinic acid production and cell growth after 48h of incubation and (B) influence of magnesium carbonate concentration on succinic acid concentration and cell growth after 96h of fermentation with (C) pH values of microbiological media before and after 96 h fermentation. Succinic acid (SA) (■); dry cell mass (DCM) (□). Fermentations were carried out in tightly capped 20-ml serum bottles with 8 ml of medium, under stirring at 160 rpm. Data are the means ±SDs from three parallel experiments. Different lower case letters designate the means with statistically significant differences ($p<0.05$).

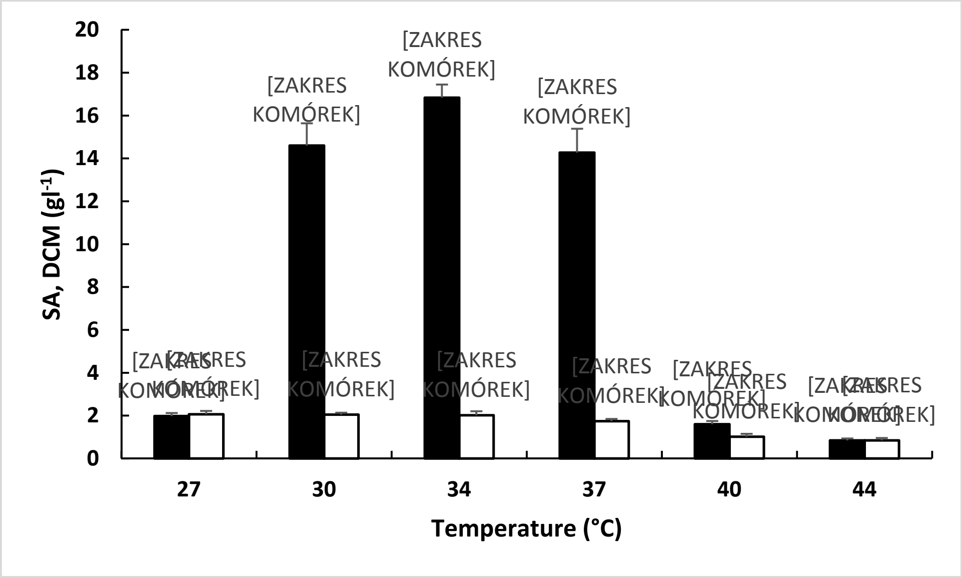

Figure S6. Effects of temperature on cell growth and succinic acid production. Data are the means ± SDs of three parallel experiments. Succinic acid (SA) (■); dry cell mass (DCM) (□). Fermentations were conducted in tightly capped 20-ml serum bottles with 8 ml of medium and stirring at 160 rpm. Data are the means ± SDs of three parallel experiments. Different lower case letters designate means with statistically significant differences ($p<0.05$).

Table S1. Sequences of oligonucleotide primers used in this study.

Primer	Nucleotide sequence (5'-3')	Reference
**27F**	5'-AGAGTTTGATCCTGGCTCAG-3'	Johnson, 1994
**1525R**	5'- AAGGAGGTGWTCCARCC -3'	
**rpoB- CM7-F**	5'- AACCAGTTCCGCGTTGGCCTG -3'	Brady et al., 2008
**rpoB- CM31b-R**	5'- CCTGAACAACACGCTCGGA -3'	
**gyr-320**	5'- TAARTTYGAYGAYAACTCYTAYAAAGT-3'	Dauga et al., 2002
**gyr-1260**	5'- CMCCYTCCACCARGTAMAGTTC -3'	

Table S2. Co-substrates tested in fermentation studies with glycerol.

Monosaccharides	Disaccharides	Sugar alcohols	Organic acids
Glucose	Lactose	Sorbitol	Glucuronic acid
Fructose	Maltose	Mannitol	Galacturonic acid
Galactose	Sucrose		Gluconic acid
Arabinose			Ascorbic acid
Ribose			Fumaric acid
Xylose			Malic acid
Mannose			Oxaloacetic acid
			Lactic acid
			Phytic acid

Table S3. Biochemical reactions of *Enterobacter* sp. LU1 in the API 50CHE system after 48h of incubation.

Tests	*Enterobacter* sp. LU1 reaction
Glycerol	+
Erythritol	-
D-Arabinose	+
L-Arabinose	+
D-Ribose	+
D-Xylose	+
L-Xylose	-
D-Adonitol	+
Methyl-ß-D-xylopyranoside	-
D-Galactose	+
D-Glucose	+
D-Fructose	+
D-Mannose	+
D-Sorbose	-
L-Rhamnose	+
Dulcitol	-
Inositol	-
D-Mannitol	+
D-Sorbitol	+
Methyl-α-D-mannopyranoside	-
Methyl-α-D-glucopyranoside	+
N-Acetylglucosamine	+
Amygdalin	-
Arbutin	+
Aesculin	+ (weak reaction)
Salicin	+
D-Celibiose	+
D-Maltose	+
D-Lactose	+
D-Melibiose	+
D-Saccharose	+
D-Trehalose	+
Inulin	-
D-Melezitose	-
D-Raffinose	+
Starch	-
Glycogen	-
Xylitol	-
Gentiobiose	+
D-Turanose	-
D-Lyxose	-
D-Tagatose	-
D-Fucose	+
L-Fucose	+
D-Arabitol	+
L-Arabitol	-
Potassium Gluconate	-
Potassium 2-Ketogluconate	+
Potassium 5-Ketogluconate	-

Table S4. Vitamin mix solution composition.

Vitamin	Concentration (mg ml^{-1})
cobalamin 0.01;	0.01
biotin 0.2;	0.2
folic acid 0.2;	0.2
thiamine 0.5;	0.5
riboflavin 0.5;	0.5
niacin 0.5;	0.5
pantothenate 0.5;	0.5
p-aminobenzoate 0.5;	0.5
lipoic acid 0.5;	0.5
pyridoxine hydrochloride 1.	1

Experimental procedures

Isolation and identification of the bacterial strain used in this study

Enterobacter sp. LU1 was isolated from the rumen of a 2-year female Saanaen goat (Bezek, Lublin Region, Poland). Its diet included green grass and maize silage. Ruminal content was diluted in solution containing 0.145 M NaCl and 0.01 M Na_2HPO_4 and was spread onto agar plates containing an isolation medium (per liter: glycerol 20 g; galacturonic acid 10g; yeast extract 5g; $MgCO_3$ 10 g; NaCl 1g; Na_2HPO_4 0.78 g; NaH_2PO_4 1.16 g; $MgCl_2$ x $6H_2O$ 0,2 g; $CaCl_2x2H_2O$ 0.2 g; hemin 10 mg; vitamin K 1 mg; cysteine hydrochloride 0.5 g; pH 7.2-7.5). The plates were incubated at 37 °C for 96 h in an Oxoid anaerobic jars with a CO_2 atmosphere. Subsequently, single isolated colonies were transferred onto new agar plates. This procedure was repeated several times to ensure culture purity. During the isolation of bacterial strains, the microbiological medium was supplemented with 1 % galacturonic acid (w/v) to improve the redox balance of microbial glycerol metabolism under anaerobic conditions. Based on the observations of the reduced character of

glycerol by Clomburg and Gonzalez (2013), combining this substrate (which has a relatively high degree of reduction) with a carbon source of lower degree of reduction (e.g. galacturonic acid, a more oxidized compound) should lead to a more balanced redox state of bacterial cells under anaerobic conditions. Thus these conditions permitted improved observation of the glycerol-utilization properties of the analysed strains. This conclusion was supported by the positive results of our studies with *Actinobacillus succinogenes* co-fermenting glycerol and galacturonic acid (improved glycerol utilization in the presence of galacturonic acid, data not shown). This approach was adopted during the search for new bacterial strains capable of succinic acid production.

A light microscope (Nikon Eclipse 90i equipped with NIS Elements AR microscope imaging software) was used for morphological examination of the newly isolated bacterial strain and cell size assumption. The *Enterobacter* sp. LU1 was deposited in International Culture Collection of Microorganisms at the Institute of Agricultural and Food Biotechnology under the number KKP 2050 (Warsaw, Poland). Strain LU1 was identified by 16S rRNA sequencing supplemented by 2 additional gene sequences, namely *rpoB* and *gyrB.* DNA primers used for amplification of analysed genes are shown in Table S1. Prior to sequencing, genomic DNA was extracted from the bacterial culture using a bacterial genomic DNA isolation kit (A&A Biotechnology, Poland). The amplified DNA fragments were purified using ExoSAP-IT (USB) and were subsequently sequenced in the Genomed Company (Warsaw, Poland). The nucleotide sequences were determined using the BigDye Terminator v3.1 Cycle Sequencing Kit (Applied Biosystems) and the capillary sequencing system 3730xl DNA Analyzer (Applied Biosystems). Basic local alignment search tool (BLAST) analysis was done to check the identity of DNA sequences in the database (Altschul

et al., 1990). Additionally biochemical characteristics of the newly isolated strain was done using API 20E and API 50CH microtests (bioMerieux, France) according to the manufacturer's recommendation. The databases at https://apiweb.biomerieux.com were used for species identification (Version 4.1).

DNA techniques

In order to amplify genes mentioned in this study, fragments of three genes were amplified by PCR. Template DNA (50 ng) and primers (final concentration 0.5 μM of each) were added to 20 μl of reaction mixture containing 200 μM of each deoxynucleoside triphosphate, 0.25 U of Taq DNA polymerase (Thermo), and PCR buffer (Thermo). Amplifications were performed with template denaturation at 94°C for 5 min, followed by 30 cycles of 94 °C for 30 s, an annealing step of 1 min at 55 °C, and extension for 2 min at 72 °C, with a final extension for 10 min at 72 °C. The primers with annealing temperatures are listed in Table S1. The PCR products were analyzed by agarose gel electrophoresis with 1.4% (w/v) agarose in a Tris-acetate-EDTA buffer (TAE). The gels were stained with ethidium bromide (0.5 μg/ml) and visualized under UV light.

Samples dillutions 1:1 with 7 % HCl (v/v) for the measuring the absorbance of the broth at 600 nm (OD_{600})

The use of hydrochloric acid ensures that the whole amount of magnesium carbonate not utilized in the process (as it is a poorly soluble in water chemical compound) will react with the acid, as a result of which magnesium chloride will be obtained that will not impede measurements of the optical density of the culture with

the spectrophotometric method. A similar methodology has been applied by scientists working on succinic acid production with the use of *Basfia succiniciproducens* (Becker et al., 2013).

Calibration curve for the biomass concentration

To construct the calibration curve, culture samples from 50 ml anaerobic cultures were added in triplicate to pre-weighed tubes and centrifuged. After a subsequent washing step with 0.9 % (w/v) NaCl, the cell pellets were dried at 80 °C for at least two days until they reached a constant mass. One unit of OD_{600} was roughly equivalent to 0.51 g l^{-1} of DCW for cells of *Enterobacter* sp. LU1 grown in BHI medium. The equation for the pre-established calibration curve was as follows:

$$DCW\ (g\ l^{-1}) = (0.45 \times OD_{600}) + 0.06$$

Nucleotide sequence accession numbers

The sequence data reported in this article have been submitted to the GenBank database under the following accession numbers:

KU499554 – partial sequence of *16S rRNA* gene

KU499555 - partial sequence of *rpoB* gene

KU499556 - partial sequence of *gyrB* gene

References

1. Altschul S.F., Gish W., Miller W., Myers E.W., Lipman D.J. (1990) Basic local alignment search tool. J Mol Biol **215**:403–410.
2. Becker, J., Reinefeld, J., Stellmacher, R., Schäffer, R., Lange, A., Meyer, H., Lalk, M., Zelder, O., von Abendroth, G., Schröder, H., Haefner, S., Wittmann, C. (2013) Systems-wide analysis and engineering of metabolic pathway fluxes in bio-succinate producing *Basfia succiniciproducens*. Biotechnol Bioeng **110**:3013-3023.
3. Brady, C., Cleenwerck, I., Venter, S., Vancanneyt, M., Swings, J., Coutinho, T. (2008) Phylogeny and identification of *Pantoea* species associated with plants, humans and the natural environment based on multilocus sequence analysis (MLSA). Syst Appl Microbiol; 31:447-460.
4. Clomburg, J.M., Gonzalez, R. (2013) Anaerobic fermentation of glycerol: a platform for renewable fuels and chemicals. Trends Biotechnol **31**:20-28.
5. Dauga, C. (2002) Evolution of the *gyrB* gene and the molecular phylogeny of *Enterobacteriaceae*: a model molecule for molecular systematic studies. Int J Syst Evol Microbiol **52**:531-547.
6. Johnson, J. L. (1994) Similarity analysis of rRNAs, pp. 683-700. In P. Gerhardt, W. A. Wood, N. R. Krieg, and R. Murray (eds.). Methods for General and Molecular Bacteriology. American Society for Microbiology, Washington, DC, USA.
